# LCK-SafeScreen-Model: An Advanced Ensemble Machine Learning Approach for Estimating the Binding Affinity between Compounds and LCK Target

**DOI:** 10.3390/molecules28217382

**Published:** 2023-11-01

**Authors:** Ying Cheng, Cong Ji, Jun Xu, Roufen Chen, Yu Guo, Qingyu Bian, Zheyuan Shen, Bo Zhang

**Affiliations:** 1 College of Pharmaceutical Sciences, Hangzhou First People’s Hospital, Zhejiang Chinese Medical University, Hangzhou 311402, China; cy028@zju.edu.cn (Y.C.); jcdq0725@outlook.com (C.J.); junjun6921@outlook.com (J.X.); 2 Hangzhou Institute of Innovative Medicine, College of Pharmaceutical Sciences, Zhejiang University, Hangzhou 310058, China; 12319021@zju.edu.cn (R.C.); yu.guo@zju.edu.cn (Y.G.); 22260233@zju.edu.cn (Q.B.); 3 Department of Pharmacy, Huzhou Central Hospital, Huzhou 313000, China

**Keywords:** LCK, off-target, ensemble machine learning, molecular docking, webserver

## Abstract

The lymphocyte-specific protein tyrosine kinase (LCK) is a critical target in leukemia treatment. However, potential off-target interactions involving LCK can lead to unintended consequences. This underscores the importance of accurately predicting the inhibitory reactions of drug molecules with LCK during the research and development stage. To address this, we introduce an advanced ensemble machine learning technique designed to estimate the binding affinity between molecules and LCK. This comprehensive method includes the generation and selection of molecular fingerprints, the design of the machine learning model, hyperparameter tuning, and a model ensemble. Through rigorous optimization, the predictive capabilities of our model have been significantly enhanced, raising test R^2^ values from 0.644 to 0.730 and reducing test RMSE values from 0.841 to 0.732. Utilizing these advancements, our refined ensemble model was employed to screen an MCE -like drug library. Through screening, we selected the top ten scoring compounds, and tested them using the ADP-Glo bioactivity assay. Subsequently, we employed molecular docking techniques to further validate the binding mode analysis of these compounds with LCK. The exceptional predictive accuracy of our model in identifying LCK inhibitors not only emphasizes its effectiveness in projecting LCK-related safety panel predictions but also in discovering new LCK inhibitors. For added user convenience, we have also established a webserver, and a GitHub repository to share the project.

## 1. Introduction

**Figure 1:**
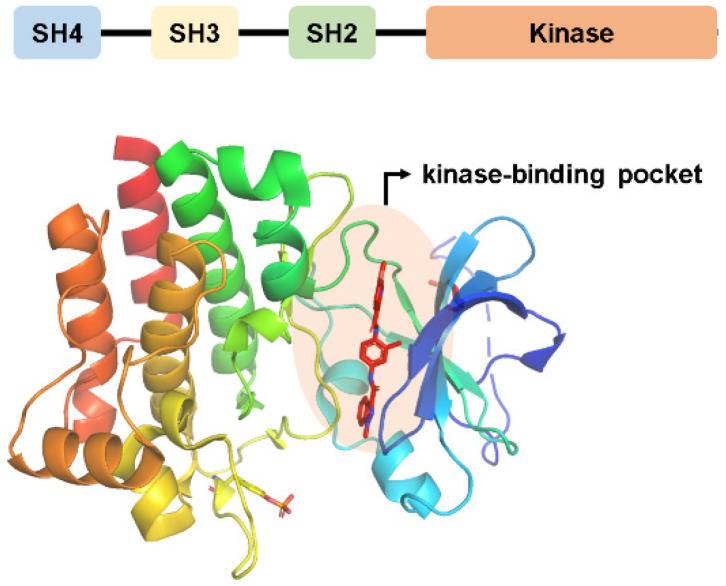
The structure and the domain detail of LCK target.

Off-target effects represent significant challenges in pharmaceutical development, frequently causing drug failures during clinical trials or provoking adverse reactions after marketing [1,2,3]. The drug off-target safety assessment panel is instrumental in evaluating these off-target effects. Its primary goal is to reduce drug-induced adverse reactions early in the development process [4,5,6]. The lymphocyte-specific protein tyrosine kinase (LCK, 56 kDa), a member of the Src kinase family, is viewed by numerous companies as a critical target to address in their safety panels [7,8]. Figure 1 provides a structural depiction of LCK, highlighting an N-terminal domain (SH4 domain), an SH3 domain, an SH2 domain, and a C-terminal tyrosine kinase structural domain. The activation of LCK is a pivotal phase in TCR signal transduction, playing a vital role in the pathogenesis of various inflammatory and autoimmune disorders such as rheumatoid arthritis, asthma, and cancer [9,10,11].

Recent research indicates that while it is a therapeutic target in various cancers, unintentional targeting can result in toxic effects [12,13,14,15]. Due to the significant homology LCK shares with other kinases in the Src kinase family, this similarity can result in off-target drug effects. Hence, there is a pressing demand for a tool that can swiftly assess the affinity of inhibitors for LCK, aiming to mitigate LCK-induced toxicity during novel drug development [7].

In recent years, advancements in database technology and artificial intelligence have significantly propelled the evolution of computer-aided drug design [16,17,18]. Consequently, there has been a surge in the development of sophisticated tools that streamline the precise evaluation of the affinity between molecular targets and drugs [17,19,20]. However, molecular docking tools often necessitate detailed structural preparation for both proteins and ligands. This complexity presents considerable challenges for novice medicinal chemists who might find it daunting to allocate the required time and expertise [21]. Conversely, AI-based QSAR [22] offers a rapid prediction of a molecule’s potential activity after model training, substantially curtailing the preparation time and lowering the experience barrier for researchers [23]. Nonetheless, a direct prediction model specifically for gauging LCK molecular affinity remains to be reported.

In this article, we introduce an open-source machine learning framework centered around a regression model. This model, which boasts a commendable R-squared (R^2^) value of 0.730 and a Root Mean Square Error (RMSE) of 0.732, is tailored to predict the binding affinity between ligands and the lymphocyte-specific protein tyrosine kinase (LCK). This framework stands as an invaluable reference for drug developers, shedding light on a molecule’s potential to inhibit LCK. Such insights guide endeavors to enhance molecular efficacy while mitigating LCK-related toxicity risks. To test the versatility of our model, we subjected it to an evaluation using the MCE-like drug library. Subsequent ADP-GLO bioassays indicated that 90% of the assessed molecules exhibited LCK-inhibitory capabilities, further cementing our model’s effectiveness. We also utilized molecular docking to shed light on the binding mechanics of these molecules. For broader accessibility, we launched an online server at (https://prod.nas.cpolar.cn/session7) (accessed on 23 October 2023).

## 2. Results and Discussion

### 2.1. Data Collaction and Preparation

**Figure 2:**
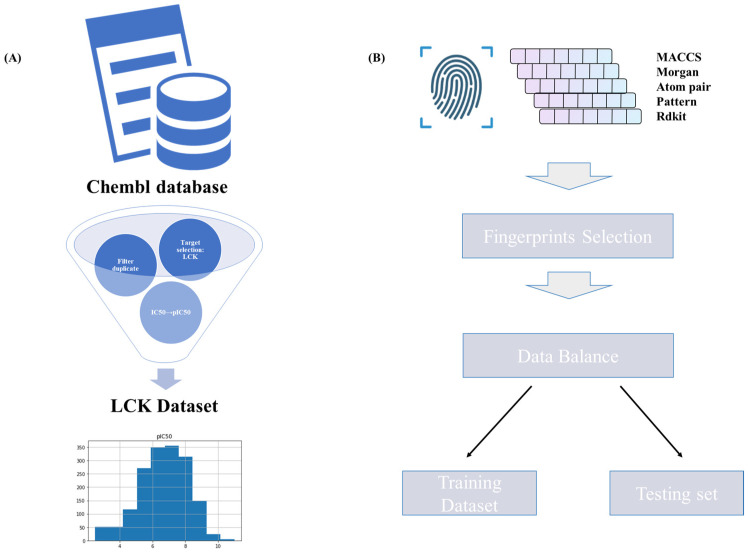
(**A**) The dataset construct process. (**B**) The fingerprint generation and the feature selection.

The workflow for data collection and preparation in our study, which outlines each curatorial step for our compound dataset, is illustrated in Figure 2. We began by procuring compound data pertinent to the LCK target from the ChEMBL database [24]. This collected dataset incorporated bioactivity data, specifically, IC_50_ values, alongside their corresponding compound structures represented in the SMILES format. To ensure data integrity, we utilized RDKit (version 2019.09.1), a comprehensive cheminformatics software, to purify and standardize the compound structures. Subsequently, we undertook data preprocessing, which encompassed the removal of missing or incomplete records. To eliminate redundancy, compounds with identical InChI keys were identified and duplicates were excised. This meticulous process yielded a curated set of 1688 unique molecules.

Subsequently, we transformed IC_50_ values into pIC_50_ values, a standard practice offering that facilitates a clearer interpretation of bioactivity data. The finalized dataset, which includes identifiers for the 1688 unique molecules, their standardized structures, and the associated pIC_50_ values, was saved in an Excel file format. This polished dataset is now ideally prepared for further analyses and modeling endeavors within our study.

### 2.2. Fingerprint Generation

To effectively characterize the molecules in our curated dataset, we employed a range of molecular fingerprinting techniques. We first tapped into RDKit’s molecular fingerprinting functionality, which yields a binary vector indicating the presence or absence of certain structural features. This method transforms the intricate three-dimensional molecular structure into a more readily interpretable one-dimensional format. Additionally, we applied atom-pair and Topological Torsions (APTT) fingerprints. Atom-pair fingerprints encode the topological relationships between atom pairs within a molecule, whereas Topological Torsions fingerprints capture the three-point correlation information [25]. Both offer nuanced insights into the molecules’ structural and topological complexities. We further utilized the Morgan algorithm to produce circular fingerprints, commonly referred to as Extended-Connectivity Fingerprints (ECFP) [26]. This algorithm crafts fingerprints by iterating over each molecule’s atoms, considering their neighborhood atoms up to a certain depth, thereby providing a more encompassing view of the molecule’s overall structure. The MACCS keys, comprising 166 predefined structural fragments, were also implemented, facilitating the rapid identification of specific substructures within molecules [27]. Finally, we employed Pattern fingerprints, often termed Daylight-like fingerprints. Rooted in the detection of distinct patterns or substructures within a molecule, these fingerprints stand out for their specificity and interpretability.

### 2.3. Fingerprint Selection

We utilized various molecular fingerprints in conjunction with a Random Forest (RF) model to predict the IC_50_ values of compounds. Initially, we divided our dataset into training and test subsets, allocating 80% of the data for training and the remaining 20% for testing. Following this partition, each fingerprinting method was employed to convert the chemical structures into numerical vectors, which were subsequently input into the machine learning model.

In parallel, we tested each fingerprint type to determine the most predictive method for our specific application. Among the array of fingerprints examined, the atom pairs and Topological Torsions fingerprints emerged as the most effective, registering a test R^2^ score of 0.699 and a test RMSE of 0.775 (Table 1).

Based on these results, we opted to prioritize the atom pairs and Topological Torsions fingerprints in the ensuing stages of our research. This decision is anchored in their superior efficacy in predicting IC_50_ values, which are pivotal to our endeavor. Through this approach, our objective is to bolster prediction accuracy, offering a robust technique for IC_50_ estimation.

### 2.4. Model Selection and Hyperparameter Tuning

In light of our updated experimental fingerprints, we began by evaluating a range of machine learning models. Our assessment encompassed a variety of models, including Random Forest (RF) [28], XGBoost, Support Vector Machine (SVM), Decision Trees (DT), K-Nearest Neighbors (KNN), LightGBM [29], AdaBoost, Gradient Boosting, Ridge, and ElasticNet. From this initial exploration, we narrowed our focus to the top-performing models for deeper refinement: Random Forest, XGBoost [30], KNN [31], and LightGBM. For each selected model, we undertook comprehensive hyperparameter tuning (Table 2).

**Figure 3:**
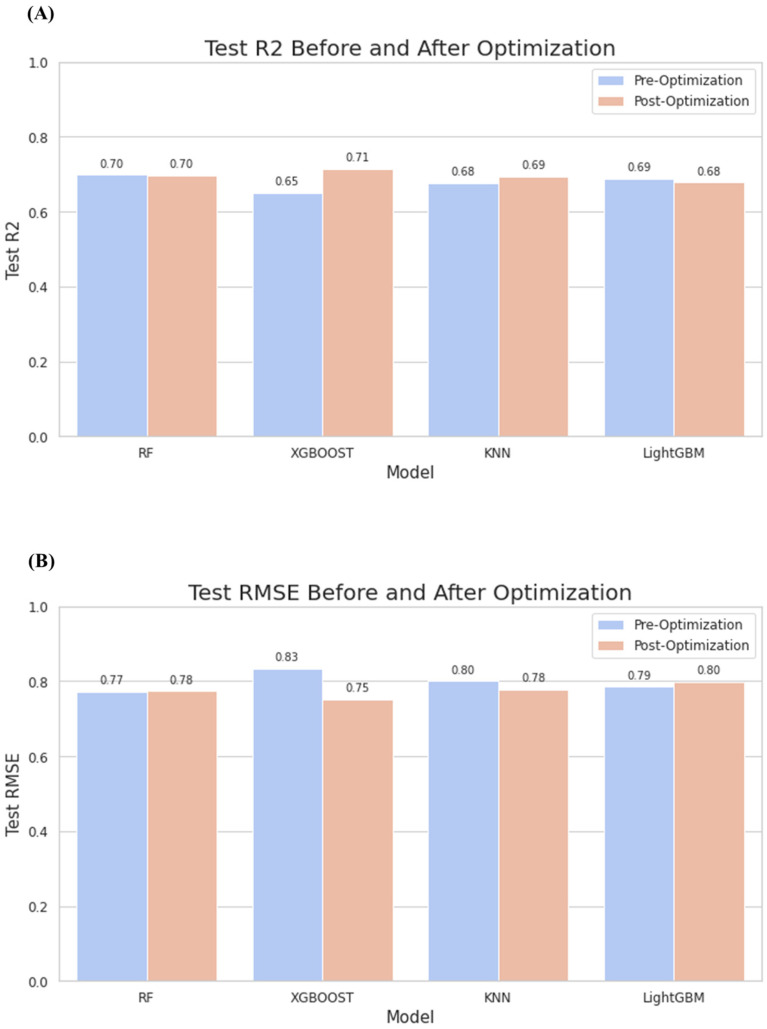
(**A**) The performance (R^2^) of the test dataset under different models before and after optimization. (**B**) The performance (RMSE) of the test dataset under different models before and after optimization.

The meticulous tuning of hyperparameters was central to enhancing our models. For this, we employed the GridSearchCV method, performing an exhaustive search across a pre-defined set of hyperparameters to find the best combinations for the Random Forest, SVM, and LightGBM models. Conversely, due to XGBoost’s extensive set of tunable parameters—which can render a full grid search infeasible—we leveraged the RandomizedSearchCV approach. This method evaluates a sample of promising hyperparameter combinations derived from defined distributions, allowing us to pinpoint the optimal configuration for the XGBoost model. Once hyperparameter optimization was complete, we retrained each model using the newly identified best parameters, evaluating their performance based on R^2^ scores and Root Mean Square Error (RMSE). This rigorous evaluation and subsequent comparison empowered us to identify the most fitting model for our upcoming molecular property prediction endeavors (Figure 3).

### 2.5. Model Ensemble

**Figure 4:**
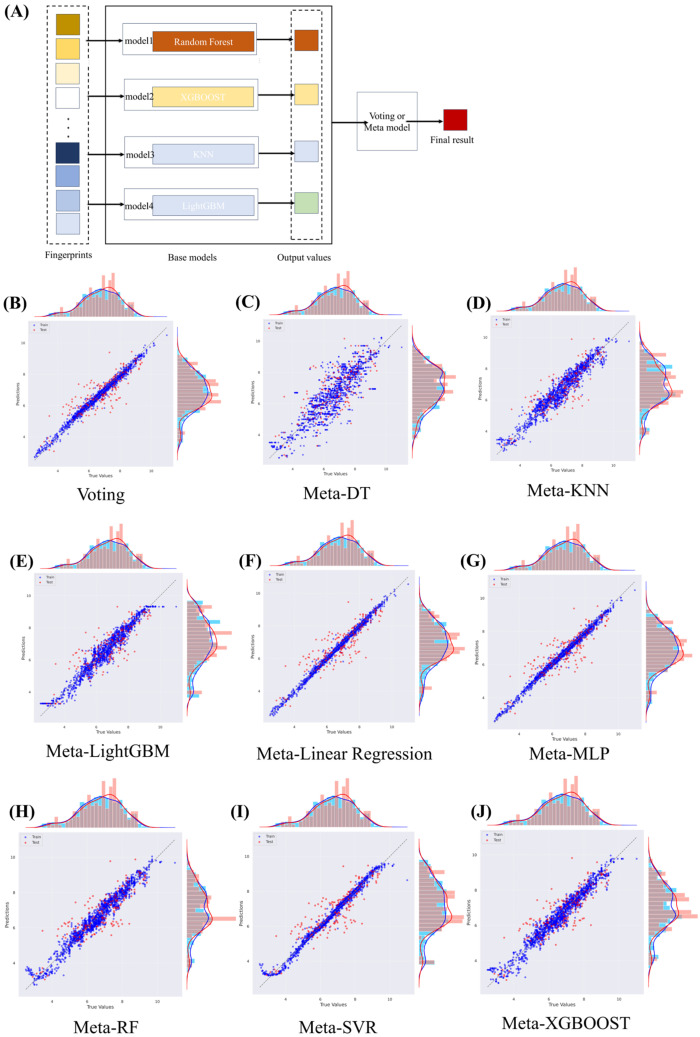
(**A**) The ensemble model structure and the performance of different ensemble ways. (**B**) Voting. (**C**) Ensemble model with a DT meta-model. (**D**) Ensemble model with a KNN meta-model. (**E**) Ensemble model with a LightGBM meta-model. (**F**) Ensemble model with a Linear Regression meta-model. (**G**) Ensemble model with a MLP meta-model. (**H**) Ensemble model with a RF meta-model. (**I**) Ensemble model with a SVR meta-model. (**J**) Ensemble model with a XGBOOST meta-model.

In our quest to develop a predictive model capable of accurately determining the binding affinity between LCK and various molecules, we embraced the principles of ensemble learning. This methodology constructs multiple individual models and combines them, with the aim of producing predictions that surpass the accuracy of any single model within the ensemble. To orchestrate this combination, we turned to the voting regressor mechanism, valued for its ability to tap into the predictive prowess of several distinct models. Our ensemble model seamlessly integrated four diverse yet synergistic base learners: Random Forest, XGBoost, KNN, and LightGBM. Each model was selected based on its outstanding performance during earlier model selection and hyperparameter tuning phases. Together, they span a broad spectrum of machine learning techniques, from ensemble methods and gradient-boosting frameworks to non-parametric approaches. This array of methodologies strengthens our ensemble model, providing a well-rounded and robust learning process. We integrated these models using a voting regressor, an ensemble meta-estimator that fits base regressors to the entire dataset and then averages individual predictions to produce a final outcome. The voting regressor uses soft voting, averaging predictions from each sub-model instead of counting a simple majority. Our methodology hinges on the assumption that these four models, given their diversity, make independent errors. Such diversity increases the chance that their errors will counterbalance each other, resulting in a more accurate overall prediction. Our aim in using a voting ensemble was to create a model that captures the strengths of each individual one, reduces variance, and bolsters the generalization capability for unseen data. The models were trained and merged using the voting regressor through the sklearn_ensemble_VotingRegressor function from the sklearn Python library. We evaluated the performance of our approach using key metrics, including the R^2^ score and Root Mean Square Error (RMSE), for both the training and testing datasets. Alongside the voting regressor technique, we also explored a stacking strategy to combine the four models. In our specific stacking implementation, we designated each of the four models as base learners, training them on the complete dataset. The preliminary predictions from these base learners were then channeled into a meta-learner, which was trained to produce the final prediction. Selecting an appropriate meta-learner is a pivotal step in stacking, as the chosen model should be adept at identifying patterns within the base learners’ predictions. We trialed various meta-learners, including DT, KNN, LightGBM, Linear Regression, MLP, RF, SVR, and XGBOOST, evaluating each based on metrics derived from a distinct validation set. Ultimately, the ensemble model that employed Linear Regression as its meta-model delivered the best performance, as indicated by an R^2^ value of 0.730 and an RMSE of 0.732. This underscores its strong predictive capacity in gauging the affinity between LCK and different molecules (Table 3, Figure 4).

### 2.6. Module’s Robust Estimator

**Figure 5:**
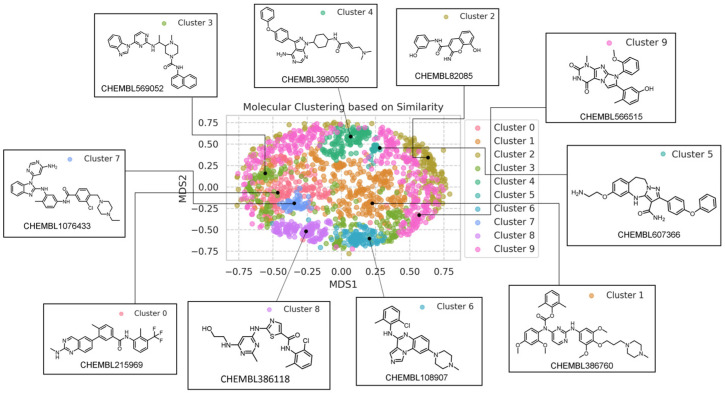
Cluster Analysis of the Model.

To validate the diversity of our dataset used for model training and testing, we employed the Multidimensional Scaling (MDS) technique, reducing dimensions based on molecular similarity (Tanimoto similarity) and categorizing the molecules into 10 clusters. As illustrated in Figure 5 it can be observed that even when the molecules are grouped into 10 clusters, their structures still display significant variation. This underscores the diversity of our training set, ensuring that our model can be applied to molecules with diverse structures.

To augment our evaluation of the models’ robustness and meticulously minimize any potential bias emanating from dataset partitioning, we judiciously applied five distinct random number seeds during the data bifurcation process, followed by a retraining of the model. Across these exacting experiments, the model garnered five sets of test R^2^ scores: 0.755, 0.744, 0.734, 0.800, and 0.748, respectively. These consistent outcomes illuminate the model’s adeptness in sustaining precise predictions across diverse dataset divisions, thereby reaffirming its reliability and general applicability.

### 2.7. Comparison with 3D-QSAR

**Figure 6:**
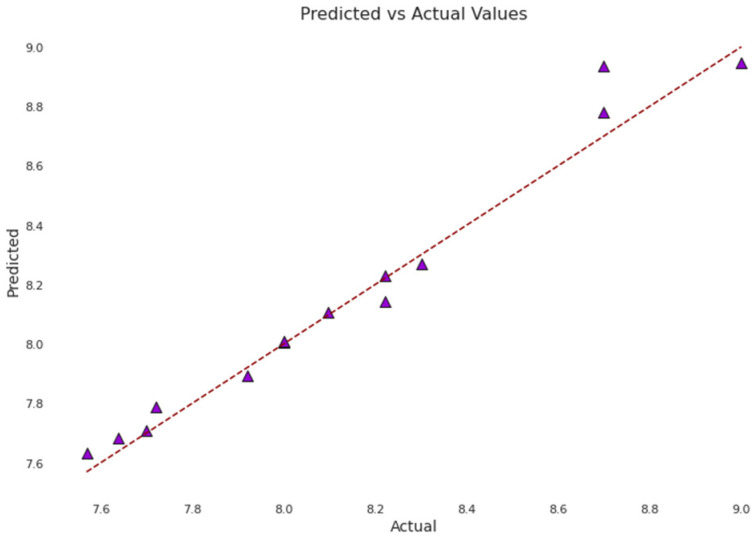
Model performance on external datasets.

In the area of QSAR analyses for LCK inhibitors, there have been notable advancements [32,33]. Until now, most current QSAR research for LCK has been through the 3D-QSAR module which leans heavily on commercial software, such as in the research by Xie et al. [33]. Concurrently, the procedures of the 3D-QSAR module are relatively intricate, and its precision is confined to molecules with analogous structures. In contrast, the model we propose is more user-friendly and versatile, catering to a variety of novel molecular frameworks. In a bid to fortify the depth and authenticity of our investigation, but hampered by licensing barriers, we incorporated the training set offered by Xie et al. [33] into our bespoke model, then proceeded to evaluate their test set. Impressively, this integration spawned significant results. As shown in Figure 6, we added the 3D-QSAR training set to our dataset and trained it in the original way. After training, we tested 3D-QSAR using its test set as our external test set. Finally, our model reports a test R^2^ score of 0.946 and a test RMSE of 0.097. This is a significant improvement over the benchmarks set by Xie et al., who reported LCK R^2^_pred values of 0.836 (CoMFA) and 0.821 (CoMSIA(SHD)), respectively [33] (Figure 6). Such a difference in outcomes accentuates the robustness of our model and potentially positions it as superior in predicting inhibitory activity.

### 2.8. Applied Modeling in LCK Novel Inhibitor Discovery

**Figure 7:**
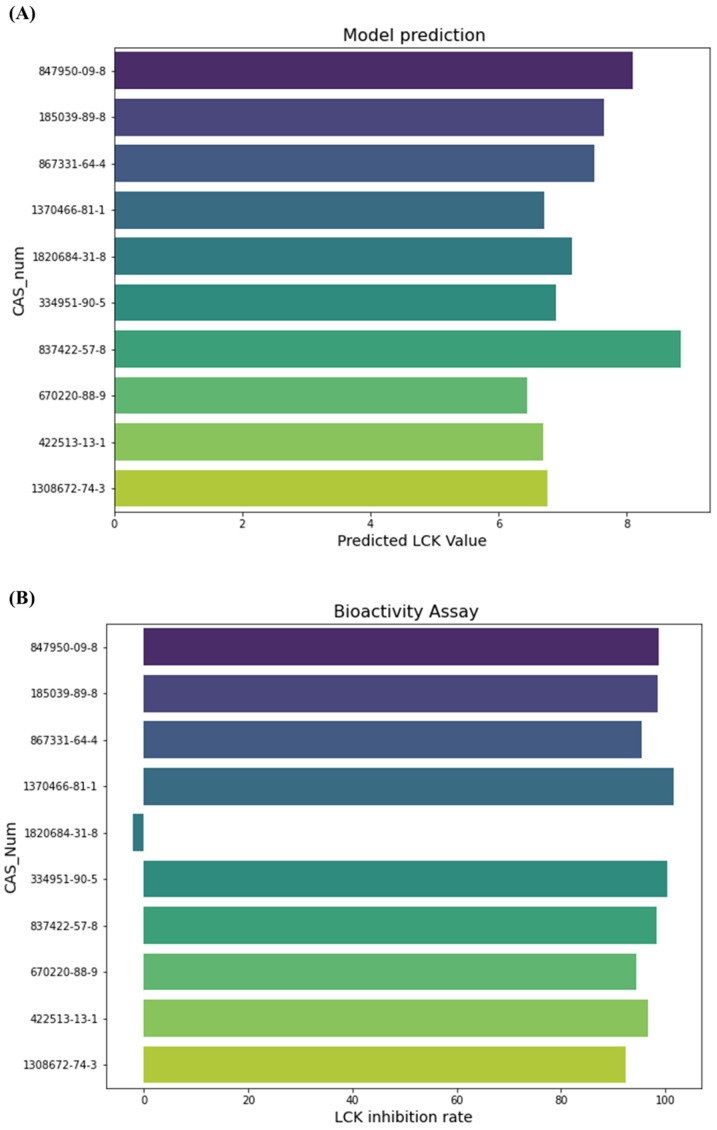
(**A**) Predicted Evaluation Scores of Top Ten Molecules for LCK Inhibition (**B**) Actual Inhibition Rates of Top Ten Molecules against LCK.

To ascertain the generalizability and practical utility of our model, we set out to identify novel LCK inhibitors. Our screening protocol included MCE-like drug libraries, consisting of approximately 9000 molecules. Using the refined model from our prior training, we predicted the activity of these molecules. Ultimately, we selected the top ten molecules, chosen due to their high evaluation scores and rational structures, for further activity tests. (Figure 7A).

In the conclusive biological assessments, a remarkable 90% of the molecules, as predicted by our model, demonstrated high inhibition rates against LCK. This outcome vividly highlights the model’s superior generalization capabilities in assessment tasks. The notable performance of these molecules is illustrated in Figure 7B. Moreover, we have launched a web server (https://prod.nas.cpolar.cn/session7) (accessed on 23 October 2023) as a convenient platform for the wider community. Here, we have made our model available, ensuring a user-friendly, accessible, and efficient tool for extended application.

### 2.9. Binding Pose Study thorough Molecular Docking

We used Deepdock, a geometric deep learning approach (https://github.com/OptiMaL-PSE-Lab/DeepDock) (accessed on 23 October 2023), to predict the binding conformations of these ten molecules with LCK [34].

**Figure 8:**
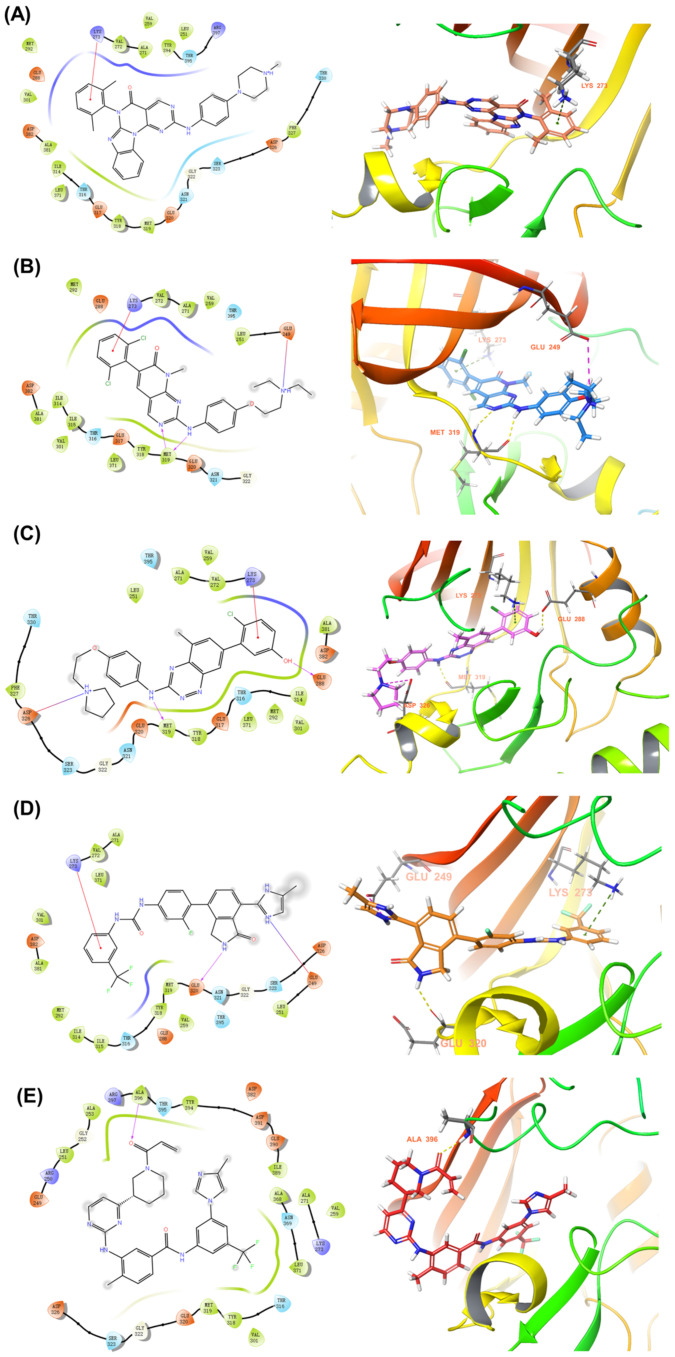

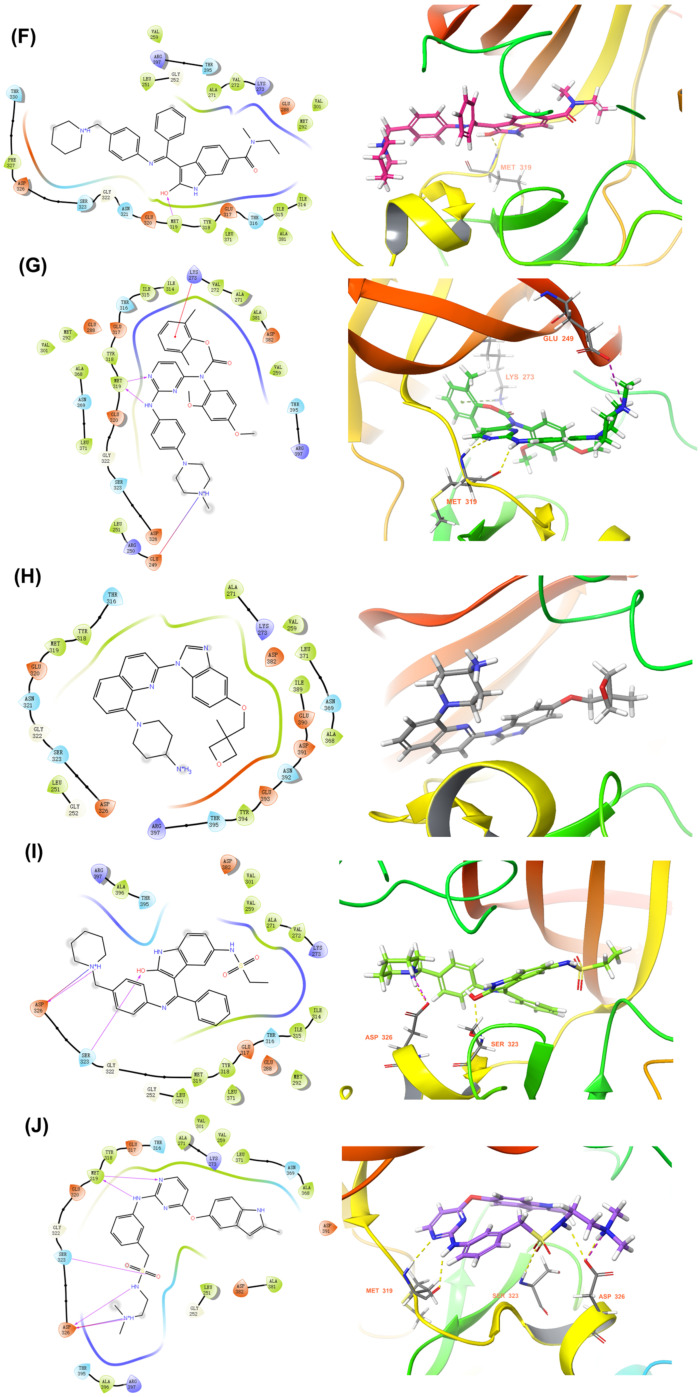
Binding modes of different molecules with LCK target. The CAS numbers of these compounds were (**A**) 847950-09-8; (**B**) 185039-89-8; (**C**) 867441-64-4; (**D**) 1370466-81-1; (**E**) 1820684-31-8; (**F**) 334951-90-5; (**G**) 837422-57-8; (**H**) 670220-88-9; (**I**) 422513-13-1; (**J**) 1308672-74-3.

Molecular docking studies showed that the *m*-xylene moiety of 847950-09-8 engages in a π–cation interaction with Lys273 (Figure 8A). Similarly, the 1,3-dichlorobenzene moiety of 185039-89-8, the 4-chlorophenol group of 867441-64-4, and the (trifluoromethyl)phenyl group of 1370466-81-1 each establish π–cation interactions with Lys273. Both the pyrimidin-2-amine of 185039-89-8 and the aniline moiety of 867441-64-4 form hydrogen bonds with Met319. The isoindole group’s -NH in 1370466-81-1 establishes a hydrogen bond with Glu320. In addition, the diethylammonium moiety of 185039-89-8 and the imidazole moiety of 1370466-81-1 could form halogen bonding interactions with Glu249. The pyrrolidin-ium moiety of 867441-64-4 also interacts with Asp326 through halogen bonding (Figure 8B–D). The 1-(piperidin-1-yl)prop-2-en-1-one moiety of 1820684-31-8 forms a hydrogen bond with Ala396 (Figure 8E). The hydroxyl group on the 1H-indol-2-ol group of 334951-90-5 and the pyrimidin-2-amine group of 837422-57-8 both interact with Met319 via hydrogen bonds. Furthermore, the *m*-xylene moiety of 837422-57-8 engages in a π–cation interaction with Lys273, while its methylpiperazinium group forms a halogen bond with Glu249 (Figure 8F,G). Interestingly, 670220-88-9 does not exhibit any inhibitory activity against LCK, possibly due to its substantial exposure to the solvent region (Figure 8H). The hydroxyl group on 1H-indol-2-ol of 422513-13-1 forms a hydrogen bond with Ser323. Both the piperidin-ium moiety of 422513-13-1 and the 2-(sulfonylamino)-*N*,*N*-dimethylethan-1-aminium moiety of 1308672-74-3 can form halogen bond interactions with Asp326. Additionally, the latter moiety forms a hydrogen bond with Ser323, and its pyrimidin-2-amine segment forms hydrogen bonds with Met319 (Figure 8I,J).

## 3. Methods

### 3.1. Data Collection

The initial dataset was sourced through the ChEMBL database API. Utilizing the pandas library in Python, we processed the data to eliminate duplicates and items missing either labels or SMILES information, ensuring optimal conditions for subsequent model training. This refined dataset contained SMILES strings—a widely-accepted notation system for molecular representation—for each molecule. Additionally, the dataset featured pIC_50_ values, derived from the original IC_50_ values. −lnIC_50_ = pIC_50_

### 3.2. Fingerprint Generation

To transform molecular structures from SMILES strings into a format amenable to machine learning analysis, we utilized the RDKit cheminformatics library to generate a variety of molecular fingerprints. These included MACCS keys, Morgan Circular fingerprints, atom-pair fingerprints, Topological Torsion fingerprints, and Pattern fingerprints. Each fingerprint type offers a distinctive perspective on molecular structure and introduces a unique feature set: MACCS keys consist of 166 predefined substructure keys. Molecules are encoded based on whether these substructures are present or absent. Morgan Circular fingerprints, also known as Extended-Connectivity Fingerprints (ECFP), are produced by iterating over each atom in the molecule and assessing its local chemical environment up to a defined radius. Atom-pair fingerprints identify specific pairs of atoms separated by a particular topological distance, offering an encompassing perspective of the molecule. Topological Torsion fingerprints, while similar to atom-pair fingerprints, also account for the path between the atom pairs, adding a richer structural context. Pattern fingerprints rely on a predefined list of SMARTS patterns, encoding whether these patterns are present or absent in the molecule.

### 3.3. Model Construction

In this study, we utilized a variety of machine learning models to construct a comprehensive ensemble model for molecular property prediction. All modeling and data preprocessing tasks were carried out in Python, leveraging several open-source libraries. Specifically, the models were developed using the Scikit-learn, XGBOOST, and LightGBM libraries.

### 3.4. Hyperparameter Tuning

Four prominent machine learning models for our predictions, Random Forest, XGBoost, KNN, and LightGBM, undertook hyperparameter tuning to optimize their performance:

Random Forest: The hyperparameters tuned in the Random Forest model included n_estimators and max_depth. n_estimators, the number of trees in the forest, was tested for the values of 100, 200, 300, 500, and 1000. The max_depth parameter, which determines the maximum depth of each tree, was tested for 10, 20, 30, and None.

KNN: The hyperparameters optimized for the KNN model included n_neighbors, weights, and metric. n_neighbors represents the number of neighbors to use for the majority-voting process and was tuned within the range of 1 to 10. The weights parameter was set to ‘uniform’ or ‘distance’, while metric was chosen from ‘euclidean’, ‘manhattan’, ‘minkowski’.

XGBoost: The hyperparameters optimized for the XGBoost model included n_estimators, learning_rate, max_depth, min_child_weight, subsample, colsample_bytree, alpha, and lambda. Each parameter was given a specific range of values to explore. The tuning process was facilitated through a Randomized Search Cross-Validation (RandomizedSearchCV) approach.

LightGBM: The hyperparameters optimized in LightGBM model included max_depth, learning_rate, n_estimators, num_leaves, and min_child_samples. Each of these parameters was given a specific range or set of options to explore.

### 3.5. Model Ensembling

Our ensemble learning approach harnessed both the voting regressor mechanism and a stacking strategy. These techniques integrated the predictive capabilities of four foundational learning models: Random Forest, XGBoost, KNN, and LightGBM.

Voting Regressor: Within the voting mechanism, each foundational model was trained individually on the entire dataset. The voting regressor was subsequently applied, utilizing a soft voting method. Rather than producing a final prediction based solely on a straightforward majority vote from the base models, this technique took the average of their individual predictions to yield the final result.

Stacking Strategy: Beyond the voting method, we incorporated a stacking technique. This required training all of the foundational models on the complete dataset, then leveraging their predictions as input features for a second level, or “meta-learner”. Selecting the appropriate meta-learner is pivotal in the stacking approach. We assessed several models for their meta-learner potential, including Linear Regression, Random Forest, Support Vector Regression, and a basic Neural Network. The efficacy of each potential meta-learner was gauged using performance metrics calculated on a distinct validation set.

Both the voting regressor and the stacking strategy techniques were executed using the sklearn.ensemble module from the sklearn Python library. Specifically, the VotingRegressor function was employed for the voting approach, while the StackingRegressor function facilitated the stacking method.

### 3.6. Molecule Docking

The protein structure of LCK, with PDB ID: 4CF3 and a resolution of 1.72 Å [35], was obtained from the RCSB Protein Data Bank [36]. It was meticulously prepared using pdb2pqr [37], which entailed determining titration states, adding any missing atoms, and attributing charges to the structure. Subsequently, residues within a 15 Å radius around the ligand were retained and featurelized via masif [38], and extraneous components were removed. The structures of small molecules underwent processing by RDKit to form 3D constructs, which were then optimized using the MMFF94 force field [39]. Docking procedures for the molecules were directly executed by Deepdock.

### 3.7. Inhibition Assay

The compound under investigation was initially dissolved in DMSO to create a 10 mM stock solution. This stock solution was subsequently diluted to produce a solution containing 50 times the final test concentrations, creating a readily accessible solution for future experiments. The assay buffer constituted 5* buffer, 5 mM MgCl_2_, 1 mM DTT, and ddH_2_O. Using this buffer, we prepared two separate solutions: a 2* ATP and substrate solution and a 2* kinase and metal solution. Using an Echo 655, we precisely pipetted 25 nL of the prepared compound into each well of a 384-well assay plate. We then added 2.5 μL of the 2* kinase and metal solution into each well and allowed the mixture to incubate for 10 min at a stable temperature of 25 °C in a polystyrene-coated 384-well assay plate. Following the initial incubation period, 2.5 μL of the 2* substrate and ATP solution was introduced into each well. The plate was subsequently incubated again at 25 °C, this time for an extended period of 50 min. We then prepared a 2* XL665 and antibody solution using detection buffer. Upon the completion of the second incubation period, we introduced 5 μL of kinase detection reagent into each well and allowed it to incubate for an additional 60 min at 25 °C. Finally, the fluorescence signals at 620 nm (Cryptate) and 665 nm (XL665) were measured using a microtiter plate reader. The resultant data were further processed using GraphPad 7.0 software, with a dose–response variable slope analysis being applied. We calculated the IC_50_ values of the tested compounds using the following formula: Y = Bottom + (Top − Bottom)/(1 + 10^((LogIC_50_ − X) ∗ hillslope)). This rigorous process ensures the highest degree of accuracy and reliability in our findings. The determination of all compounds was carried out at a concentration of 25 μM, and column homology was generated by seaborn library.

### 3.8. Webserver Construction

The structure of our web-based solution is bifurcated into two essential components: the front end and the back end. The front end, designed with CSS and Bootstrap, facilitates a responsive and intuitive user interface. Conversely, the back end, architected with Python’s Django framework, undertakes significant computational functions. A predictive model, embedded within the back end, employs pre-trained parameters that have been loaded into the system. This model is equipped to execute predictions on incoming data structured in the Simplified Molecular Input Line Entry System (SMILES) format.

A notable benefit of this methodology is the system’s ability to perform real-time forecasts, eliminating the necessity for the model to repeatedly learn with each new SMILES file received. As such, this configuration optimizes the efficiency and accuracy of predictions, thereby enhancing the overall functionality of the webpage.

## 4. Conclusions

In this study, we developed a predictive model for the discovery of novel LCK inhibitors. Our comprehensive approach encompassed data collection and preprocessing, fingerprint generation and selection, model selection and hyperparameter tuning, a model ensemble, and its ultimate application in drug discovery. Our fingerprint choices, atom pairs and Topological Torsions, demonstrated exceptional capability in predicting IC_50_ values. Among the variety of machine learning models evaluated, Random Forest, XGBoost, KNN, and LightGBM consistently outperformed the rest. To ensure robustness and augment generalizability, we incorporated ensemble learning into our methodology. We amalgamated the chosen models using a voting regressor mechanism along with a stacking strategy. The efficacy of these ensemble models was assessed through the R^2^ score and Root Mean Square Error (RMSE) across both the training and testing datasets. To deepen the breadth and fortify the credibility of our investigation, we merged the dataset from Xie et al. into our model. After this integration, our findings were substantial. Specifically, our model registered an R^2^ score of 0.946 and a test RMSE of 0.097, denoting a significant improvement over the metrics presented by Xie et al. [33]. The predictions from our ensemble models consistently outperformed those of the individual constituent models, underscoring the efficacy of our approach. Utilizing these models, we screened an MCE-like drug library, leading to the identification of ten promising LCK inhibitors. Subsequent biological testing via the ADP-Glo kinase assay confirmed that nine of these exhibited definitive LCK inhibitory activity, highlighting the practical utility of our model. In summary, our research offers a potent model for LCK inhibitor discovery, emphasizing the transformative role of machine learning in optimizing drug discovery processes. Moreover, we have introduced a user-friendly web server for effortless accessibility. Looking ahead, we aim to expand our model to encompass other relevant factors and to subject the predicted inhibitors to thorough preclinical and clinical evaluations.

## Figures and Tables

**Table 1:** The performance of each fingerprint under Random Forest model.

	Training R^2^	Test R^2^	Training RMSE	Test RMSE
RDKit	0.957	0.671	0.308	0.808
APTT	0.954	0.697	0.319	0.775
Morgan	0.956	0.65	0.314	0.834
MACCS	0.929	0.644	0.397	0.841
Pattern	0.954	0.650	0.320	0.834

**Table 2:** The performance of different models adapting the APTT fingerprint.

	Training R^2^	Test R^2^	Training RMSE	Test RMSE
RF	0.954	0.699	0.319	0.773
XGBOOST	0.997	0.65	0.088	0.834
SVR	0.805	0.631	0.66	0.856
DT	0.999	0.327	0.006	1.156
KNN	0.773	0.677	0.71	0.801
LightGBM	0.962	0.689	0.291	0.786
AdaBoost	0.536	0.486	1.016	1.01
GradientBoosting	0.536	0.486	1.016	1.01
Ridge	0.973	0.04	0.246	1.38
ElasticNet	0	−0.007	1.492	1.414

**Table 3:** The performance of different ensemble methods.

	Test R^2^	Test RMSE
Voting	0.722	0.743
Meta-LinearRegression	0.730	0.732
Meta-RandomForest	0.692	0.781
Meta-SVR	0.725	0.738
Meta-MLPRegressor	0.725	0.738
Meta-LightGBM	0.695	0.778
Meta-XGB	0.691	0.782
Meta-KNN	0.683	0.793

## Data Availability

The data referenced in the paper, along with the final model’s code and weights, can be found at the following link: https://github.com/shenzheyuan2020/LCK_QSAR (accessed on 23 October 2023).
